# Cloning and Characterisation of Multiple Ferritin Isoforms in the Atlantic Salmon (*Salmo salar*)

**DOI:** 10.1371/journal.pone.0103729

**Published:** 2014-07-31

**Authors:** Jun-Hoe Lee, Nicholas J. Pooley, Adura Mohd-Adnan, Samuel A. M. Martin

**Affiliations:** 1 Institute of Biological and Environmental Sciences, University of Aberdeen, Aberdeen, United Kingdom; 2 School of Biosciences and Biotechnology, Faculty of Science & Technology, University of Kebangsaan, Selangor, Malaysia; 3 Malaysia Genome Institute, Ministry of Science, Technology and Innovation, Selangor, Malaysia; INRA, France

## Abstract

Ferritin is a highly-conserved iron-storage protein that has also been identified as an acute phase protein within the innate immune system. The iron-storage function is mediated through complementary roles played by heavy (H)-chain subunit as well as the light (L) in mammals or middle (M)-chain in teleosts, respectively. In this study, we report the identification of five ferritin subunits (H1, H2, M1, M2, M3) in the Atlantic salmon that were supported by the presence of iron-regulatory regions, gene structure, conserved domains and phylogenetic analysis. Tissue distribution analysis across eight different tissues showed that each of these isoforms is differentially expressed. We also examined the expression of the ferritin isoforms in the liver and kidney of juvenile Atlantic salmon that was challenged with *Aeromonas salmonicida* as well as in muscle cell culture stimulated with interleukin-1β. We found that each isoform displayed unique expression profiles, and in certain conditions the expressions between the isoforms were completely diametrical to each other. Our study is the first report of multiple ferritin isoforms from both the H- and M-chains in a vertebrate species, as well as ferritin isoforms that showed decreased expression in response to infection. Taken together, the results of our study suggest the possibility of functional differences between the H- and M-chain isoforms in terms of tissue localisation, transcriptional response to bacterial exposure and stimulation by specific immune factors.

## Introduction

Iron is a vital trace element that functions as a biocatalyst or electron carrier for many biological reactions such as energy metabolism, cell proliferation and immunity [1], [2]. Iron is thus an essential resource for almost all organisms, including pathogens that require iron for proliferation and production of virulence factors (except *Borrelia burgdorferi*, the causative agent of Lyme disease) [3], [4]. Therefore, iron metabolism is closely linked with the innate immune response, in which the host attempts to limit pathogen access to iron through an iron-withholding strategy [5]. A general approach of this strategy entails the suppression of iron efflux from iron-storage cells (e.g. macrophage and duodenal enterocytes), followed by the increased accumulation of iron within cells [6]. Further support for the hypothesis of the iron-withholding strategy came from previous studies that found many of the upregulated plasma proteins during the acute phase response (APR) are also involved in iron metabolism [6], [7]. These plasma proteins are termed as positive acute phase proteins (APP) and examples include ferritin, hepcidin and transferrin.

Ferritin is a highly-conserved protein that sequesters excess iron into a non-toxic and biologically-available form [8]. Careful regulation of iron is essential as unbound iron triggers the formation of free radicals that damage cellular lipids, proteins and nucleic acids [2]. Iron storage involves two major step – the oxidisation of Fe(II) followed by transport and mineralisation into a stable iron core [8]. Both these steps are carried out by the heavy (H) and light (L)-chains respectively in tetrapods, hence the apoferritin structure that consists of 24 subunits of both chains. The ratio of H/L-chains in a ferritin molecule exhibits spatio-functional variations. For example larger proportions of L-chains are found in isoferritins in liver tissue while the H-chains are more predominant in heart tissues that are involved in rapid iron exchange [8].

The expression of vertebrate ferritin is regulated at both the transcriptional and translational level. Translational control of ferritin is mediated by a conserved RNA structure in the 5′-untranslated terminal (UTR), known as the iron-responsive element (IRE) [9]. The IREs provide a binding site for the iron-binding proteins, which dissociate from the IRE in high iron conditions and permit the translation of the ferritin mRNA. It is hypothesised that the translational control of ferritin is more responsive towards changes in iron levels, whereas the transcriptional control is linked to oxidative stress, inflammation and immunity [10]. Gene expression of H-chain is increased by interleukin (IL)-6 and tumour necrosis factor (TNF)-α, while IL-1β has a positive effect on both transcription and translation of the H-chain [11]. On the other hand, IL-1β and TNF-α have minimal effect on the L-chain transcription, though the latter led to increased L-chain translation [10], [11].

In contrast to their mammalian counterparts, ferritin from other vertebrates has not been as well-studied. A third ferritin subunit named as the middle (M)-chain was identified along with the H- and L-chains in bullfrog (*Rana catesbiana*) [12]. The first report of ferritin in fish was from the Atlantic salmon (*Salmo salar*), in which two ferritin subunits H- and M-chains were described [13]. Subsequent studies of ferritin in various teleosts such as dusky rockcod (*Trematomus newnesi*) [14], Croceine croaker (*Pseudosciaena crocea*) [15] and turbot (*Scophthalmus maximus*) [16] reported similar findings on both the H- and M-chains. The M-chain is capable of carrying out both the requisite steps in iron storage, as it possess the conserved ferroxidase centres of H-chain and carboyxl ligands of L-chains [17]. We previously carried out extensive *in silico* analysis on vertebrate ferritins and found that the teleost M-chain is orthologous to the mammalian L-chain [18]. In addition, we also observed that several teleost species such as zebrafish (*Danio rerio*) possess several ferritin isoforms, consistent with the proposed whole genome duplication events in teleost [19]. However, thus far there has only been one report of multiple H-chain ferritin isoforms, found in rainbow trout (*Oncorhynchus mykiss*) [20].

In this study, we report for the first time the isolation of multiple ferritin isoforms from both the H- and M-chains in a vertebrate species. We identified five ferritin isoforms (H1, H2, M1, M2, M3) in the Atlantic salmon through gene structure and phylogenetic analyses. We also analysed the expression of the multiple isoforms in infected *S. salar* and IL-1β stimulated cells, and observed unique expression profiles for the various ferritin isoforms. For clarification purposes, in this article we use the term ‘subunit’ to refer to the distinct H- or M-chains and ‘isoform’ refers to the individual copies of the H- and M-chain groups.

## Materials and Methods

### Ethics statement

All animals were handled in strict accordance with UK legislation on scientific procedures on living animals. The protocol was approved by the ethics committee at University of Aberdeen and the work was carried out under the project licence number PPL 60/4013.

### RNA extraction for sequence generation and tissue distribution

Tissue samples of trunk kidney, head kidney, liver, muscle, brain, gills and intestine were extracted from four juvenile mixed sex Atlantic salmon (approximately 40 g) maintained in freshwater aquarium facilities, University of Aberdeen. The water conditions were kept at a constant 12°C, pH 7.60 (±0.05) and 90% (±1%) of oxygen saturation, and the fish were fed *ad libitum* (Nutrico feed). Fish were killed by schedule 1 method which was overdose of anaesthetic followed by destruction of the brain and tissues stored in RNAlater (Ambion) at 4°C for 23 hours followed by storing at −80°C until RNA extraction. Total RNA was isolated with TRIZol (Invitrogen) from 100 mg of tissue that was homogenised using tungsten carbide beads (3 mm, Qiagen), following the manufacturer's instructions. Total RNA was estimated using Nanodrop (Agilent Technologies) and the RNA integrity was assessed quantitatively with the Bioanlayser 2100 (Agilent Technologies), RNA was assessed as being high quality if the 28S peak was equal or greater than 18S peak. The RNA was then stored at −80°C until required for cDNA synthesis.

The synthesis of cDNA was carried out using BioScript reverse-transcriptase (Bioline) and oligo-dT primers, with approximately 1 µg of total RNA used for each sample. The resulting cDNA was then diluted to a final volume of 50 µl 1× TE buffer and stored at −20°C.

### Generation of complete ferritin coding sequences

A search for sequences similar to ferritin was conducted against the NCBI GenBank database. The retrieved sequences were then compared with EST records to predict the presence of the iron-responsive element (IRE) in the 5′-untranslated regions (UTR). To obtain the complete coding sequences, flanking primers were manually designed in the 5′- and 3′-UTR regions respectively (Table 1). Polymerase chain reaction (PCR) was carried out on cDNA generated from liver tissues with the following parameters: 95°C for 20 s, 35 rounds at 57–60°C for 30 s, 72°C for 2 min and an additional extension at 72°C at 10 min.

**Table 1:** List of PCR primers used in this study.

Gene		Primer sequence (5′ to 3′)
***PCR***		
FerH	F	CGTCAAGAAACCAGAGAAGGA
	R	AGGTAGTGGGTCTCAATGAAGTC
FerM1	F	GTAGCAAAATAGTCGGAGGAAC
	R	CCCCTCCCTATAAATGCAAAGC
FerM2	F	CGTAACACTTACTTGAACTGTCT
	R	CCTCCAATACAATAGTGTTGTCAAC
FerM3	F	ACGTAACACTTACTTGAACTCTC
	R	CCTTGCCTCCAAAATACAATAG
***quantitative PCR***		
*ef1α*	F	CAAGGATATCCGTCGTGGCA
	R	ACAGCGAAACGACCAAGAGG
*βact*	F	TGACCCAGATCATGTTTGAGACC
	R	CTCGTAGATGGGTACTGTGTGGG
FerH	F	CGTCAAGAAACCAGAGAAGGA
	R	AGGTAGTGGGTCTCAATGAAGTC
FerM1	F	ATCCGCCAGAACTATCACCA
	R	CTGGCTTCTTGATGTCCTGG
FerM2	F	AAATGAAGTCTCAGGTCCGC
	R	TGTCCTGAAGGACAATGCGT
FerM3	F	TGGAGATGTTTGCTTCTTATACC
	R	CTTTCTGGCTTCGTGATGTC

PCR products were analysed by agarose gel electrophoresis and were ligated into the pGEM-T Easy vector (Promega) prior cloning into JM109 competent cells (ActiveMotif), according to the manufacturer's protocols. Plasmid extractions were performed using the QIAPrep Spin Miniprep Kit (Qiagen) and sent for sequencing to Eurofins MWG Operon.

### Sequence analysis

The nucleotide sequence data were screened for vector regions with VecScreen, followed by the prediction of the open reading frames (ORF) using the ORF Finder analysis tool (http://ncbi.nlm.nih.gov/projects/gorf). Subsequently, nucleotide BLAST searches were conducted against the NCBI GenBank to retrieve the respective full-length EST sequences. The EST sequences were used to predict the presence of IREs in the 5′-UTR using the SIREs web server tool (http://ccbg.imppc.org/sires/index.html) [21].

### Phylogenetic analysis

For phylogenetic analysis, only manually annotated protein sequences from teleosts and mammals were retrieved from UniProtKB/Swiss-Prot (www.uniprot.org/), due to many sequences being incorrectly annotated in NCBI GenBank due to the high similarity between the various ferritin subunits (Table S1). Sequences were also retrieved from the Ensembl database for species that possessed more than two copies of the ferritin gene including *D. rerio*, *Gasterosteus aculeatus* and *Xenopus tropicalis* [18]. The ferritin sequence from lamprey (*Petromyzon marinus*) was also retrieved from GenBank to be used as an outgroup representing an ancestral chordate [22]. Multiple sequence alignment was carried out with MAFFT (version 6.864b) software using the L-INS-i alignment strategy [23]. This was followed by phylogenetic analysis using Maximum-Likelihood as implemented by PHYML (version 3.0) [24]. The LG model was selected with optimised gamma distributions and proportion of invariant sites, followed by non-parametric bootstrap tests of 1000 replicates [24], [25].

### Codon substitution analysis

Nucleotide sequences were obtained from NCBI Genbank and Ensembl for every protein sequence, with the exception of five sequences from *T. newnesi and Trematomus. bernacchii* in which no nucleotide sequences were available. The ORFs in each nucleotide sequence were then used to generated a codon alignment based on the protein alignment, using the reverse-translate feature in trimAL [26]. The codon alignment was then analysed with the yn00 utility in PAML to estimate non-synonymous (*d_N_*) and synonymous substitution rates (*d_S_*) between every pairwise sequence using the Yang and Nielsen (2000) method [27], [28].

### Bacterial challenge

Juvenile Atlantic salmon were maintained as previously described for RNA extraction. The fish were then anaesthetised with bezocaine (Sigma 20 mg/l) and injected intraperitoneally with 100 µl of a genetically attenuated (aro A-) strain of *Aeromonas salmonicida* (Brivax II) (10^9^ CFU/ml) in PBS or 100 µl of PBS as control [29]. Fish were sacrificed at 24 h following the experimental infection and total RNA was extracted from liver and kidney tissues using similar methods as previously described. A total of 48 fish were used, with half of the amount infected with *A. salmonicida* and the remaining half were maintained as control.

### Myosatellite isolation, preparation and stimulation with interleukin 1β

Atlantic salmon (mean weight of 25 g and mean length of 12 cm) were used for skeletal muscle myosatellite cell extraction, as previously described [30]–[33]. For each muscle extraction 6 fish were used (∼1.5 g tissue from each fish), this was to remove any individual fish effects. Prior to plating cells on 6 well plates, laminin (mouse laminin, Sigma-Aldrich) was applied to the well surfaces 24 h before the cells were plated out, at a concentration of 1 mg/ml. Cell cultures were then left for 1 h for microsatellite cells to bind to the surface before the medium (Leibovitz L15 medium (Gibco) + penicillin/streptomycin 1% (Pen/Strep, Gibco, Penicillin 10,000 units/ml, streptomycin 10,000 µg/ml)) was first changed and cells allowed to differentiate at 22°C, with the medium being changed every 2 days. Following 4 days growth the medium was removed and 1 ml of new medium (with 0.5% FCS) containing either 10 µl recombinant trout IL-1β protein (rIL-1β) to achieve a concentration of 25 ng/ml or cell were non-stimulated as control with 10 µl PBS. RNA was extracted as described above.

### Gene expression by quantitative PCR

Gene-specific primers that spanned the exon boundaries were designed for the various ferritin isoforms (H1, H2, M1, M2, M3) using the PerlPrimer software (v1.1.19) based on the complete coding sequence obtained earlier (Table 1) [34]. Due to the high similarity between the H1 and H2 sequence (98% similarity) and subsequent difficulty in designing specific primers to distinguish between them, the amount of H-chain transcripts was measured as the total combination of H1 and H2 transcripts.

The reaction set-up consisted of 10 µl of 2× GoTaq qPCR Mastermix (Promega), 3 µl of cDNA and 0.1 µl of each primer and finalised with nuclease-free water (Fisher) to a total of 20 µl. The control and treated samples were analysed simultaneously in a 96-well plate on the DNA Engine Opticon system (MJ Research, Inc.) with the following programme: 95°C for 5 min, then 40 cycles of 94°C for 30 s, 62°C for 30 s, 72°C for 30 s, 79.5°C for 30 s (reading of plate), and melting curve analysis between 72°C and 94°C to determine the specificity of the amplicons. Negative (no-template) controls were also run simultaneously for each amplicon group. Four biological replicates (n = 4) were used for the tissue distribution and IL-1β-stimulated-cell culture analysis, while eight (n = 8) biological replicates were used for bacterial challenge analysis. For the bacterial challenge analysis, samples were selected from individual that showed significantly elevated levels of serum amyloid A in the liver by real time PCR (>450 fold-change, data not shown), which indicated the presence of a strong acute phase response [35].

For data analysis, the expression data obtained for tissue distribution and bacterial challenge were normalised to the housekeeping genes elongation factor-1α (*ef1α*) and β-actin (*βact*), as the expression stability of both genes have been demonstrated in previous studies [36]. However, for the expression analysis in IL-1β stimulated-muscle cells, only *ef1α* was used as *βact* was reported as an unsuitable reference gene in myogenic cell cultures [33]. The efficiency of the amplification reaction for each amplicon group was determined using a 10-fold dilution series that was run simultaneously with the experimental samples. The efficiency was calculated according to the formula E = 10^(−1/s)^ where *s* is the slope generated from the dilution series, with the log dilution plotted against Ct (threshold quantification cycle).

The Pfaffl method was used to calculate the fold change in expression of ferritin in the bacterial challenge and IL-1β stimulated [37]. Unpaired *t*-tests were then conducted, in which p-values<0.05 were considered to indicate significant differences between the control and infected samples. The expression data were finally presented as mean ± standard error.

## Results

### Identification and analysis of multiple ferritin isoforms

Five unique coding sequences of various sizes were obtained through PCR, which were named ferritin H1 (641 bp) (JX480494), H2 (638 bp) (JX480495), M1 (694 bp) (JX480496), M2 (778 bp) (JX480497) and M3 (788 bp) (JX480498). Subsequent nucleotide BLAST searches against the NCBI GenBank retrieved the following matches with ≥99% similarities: BT060404.1 (H1), BT060211.1 (H2), BT050033.1 (M1), BT048838.2 (M2) and BT046816.2 (M3). Each of these sequences retrieved from GenBank were annotated as ferritin genes in *S. salar* and possess IREs in their respective 5′-terminals as predicted by the SIREs web server. The IRE sequences were identical between H1 and H2, as well as between M1, M2 and M3 (Figure S1). All ferritin isoforms contain an identical UGC bulge. However, there are differences observed for the apical loop sequence between the H- and M-chain isoforms, respectively. The canonical loop sequence CAGUGC is maintained in the H-chain isoforms, but the cysteine residue at the sixth position was substituted with adenine (CAGUGA) in the M-chain isoforms.

**Figure 1:**
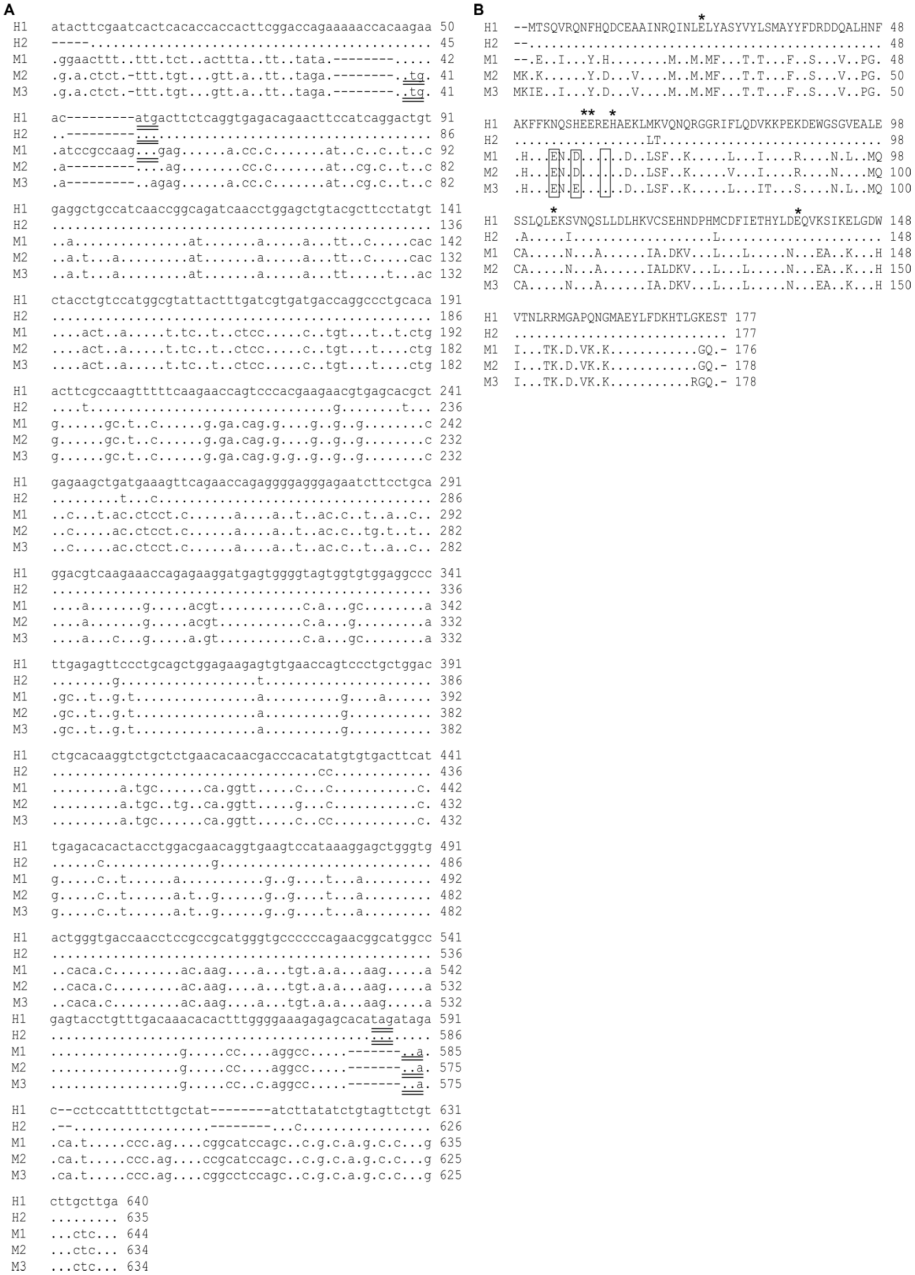
Nucleotide sequences of the Atlantic salmon ferritin cDNAs A) Nucleotide sequences of the Atlantic salmon ferritin cDNA clones H1, H2, M1, M2 and M3. Identical nucleotide residues are indicated by periods, while substituted residues are shown. The start and termination codons are underlined. B) Amino acid sequences based on the predicted ORFs of H1, H2, M1, M2 and M3. Identical amino acid residues are indicated by periods and substituted residues are shown. The conserved ferroxidase centres and nucleation sites are respectively indicated by * and boxes.

An ORF of 534 bp was predicted for H1 and H2 respectively and M1 was predicted to possess an ORF of 531 bp. Both M2 and M3 were respectively predicted to contain an ORF of 537 bp. Comparisons of the UTR between H1 and H2 showed 99% similarity, while the UTR of M1 share 99% with M2 and 97% with M3, respectively (Figure 1A). A high degree of similarity was also observed for the amino acid sequences, with H1 and H2 sharing 97% similarity and M1 sharing 96% similarity with both M2 and M3 (Figure 1B).

### Multiple sequence alignment

A multiple sequence alignment was generated using the amino acid sequences ferritin subunits from various teleost, amphibian and mammalian species to compare the presence of conserved ferroxidase centre and nucleation residues. Analysis of the alignment showed that both H1 and H2 possess the conserved ferroxidase centre residues (Glu24, Glu58, Glu59, His62, Glu104, Gln138) that are also found in mammalian H-chains (Figure S2). The ferritin isoforms M1, M2 and M3 possess the conserved ferroxidase center residues as well as two conserved nucleation residues observed in the mammalian L-chain (Glu54, Glu61). However, the second nucleation residue (Glu54) is observed to be less conserved among the teleost M-chains and is substituted with Asp in M1, M2 as well as in other species such *P. crocea* [15], *T. bernachii* [17], *O. mykiss* [20] and *Sciaenops ocellatus* [38].

### Analysis of gene organisation

**Figure 2:**
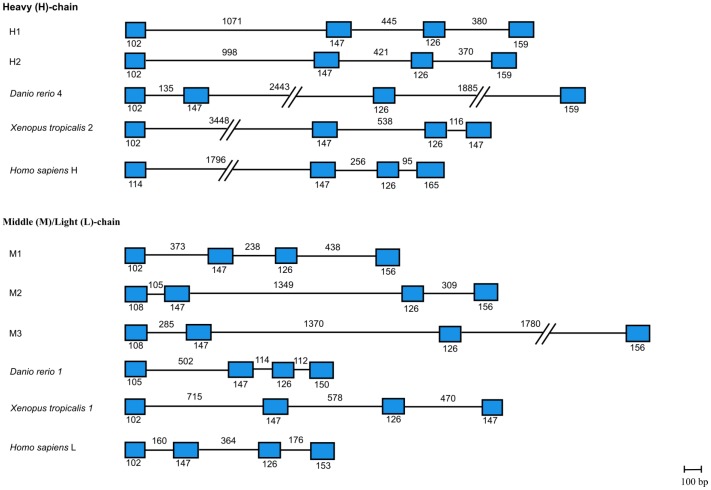
Comparison of the gene organisation of H1, H2, M1, M2 and M3 from *S. salar* with ferritin sequences from other vertebrates. Exons and introns are represented respectively by the blue boxes and black lines. Numbers below and above the boxes respectively indicate exon sizes and intron sizes. The length of exons and introns is drawn to scale except for intron sizes exceeding 1500**//**.

The gene organisation of the ferritin isoforms in *S. salar* were predicted through best-BLAST hits against the whole genome shotgun contigs in the NCBI GenBank database: H1 (AGKD01130893.1), H2 (AGKD01000812.1), M1 (AGKD01034433.1), M2 (AGKD0100120.1) and M3 (AGKD01000014.1). All isoforms possess an identical 4-exons/3-introns structure, consistent with ferritins from other vertebrates [8] (Figure 2). The size of each exon was highly similar across vertebrates, with the size of the second and third exon being conserved across various vertebrate species that were examined.

### Phylogenetic analysis

**Figure 3:**
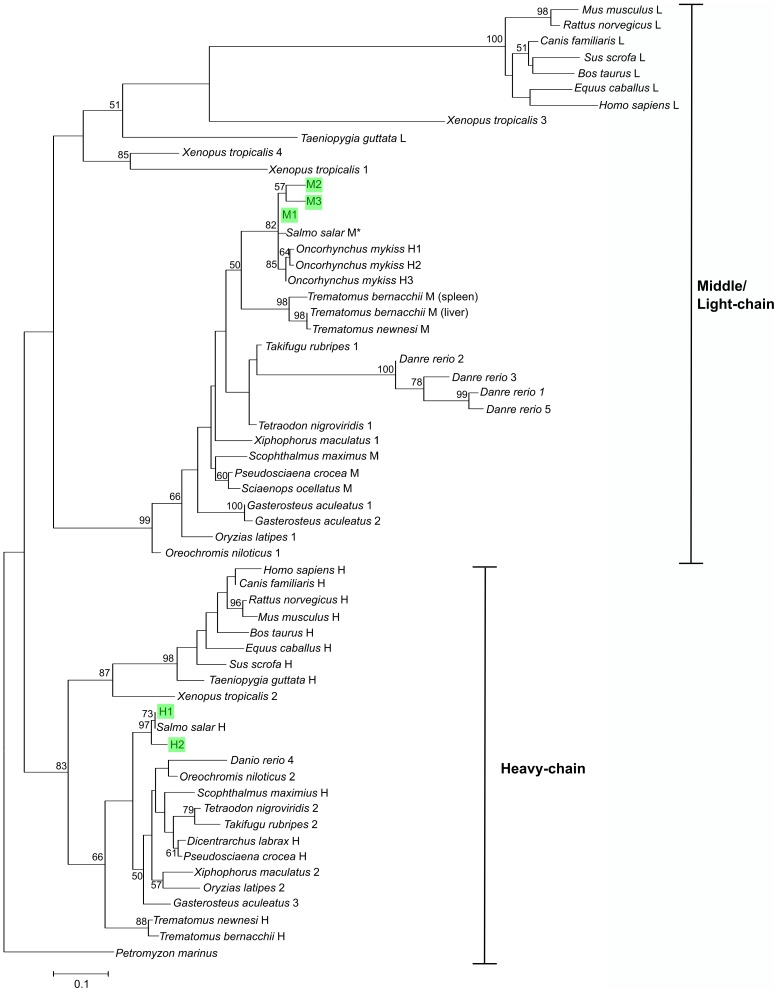
Maximum Likelihood tree generated from amino acid sequences of vertebrate ferritins. The various ferritin sequences are clustered according to chain types, with heavy(H)-chains forming an orthologous group to the middle (M)/light(L)-chains. Ferritin sequences from *S. salar* (H1, H2, M1, M2, M3) characterised in this study are highlighted in green, while previously reported sequences [13] are indicated with (*). Values at nodes indicate the Maximum-Likelihood bootstrap percentages (1000 replications). The scale bar represents the estimated number of substitutions per site.

A phylogenetic tree was constructed with the Maximum Likelihood algorithm using PhyML 3 (Figure 3). Due to the presence of ferritin pseudogenes and various ferritin subunits with inaccurate annotations, only published sequences were used for the phylogenetic analysis [39]. The phylogenetic tree shows a clear clustering of sequences according to chain types. The H-chain sequences from teleosts, amphibians and mammals were observed to cluster together. On the other hand, the teleost M-chain and tetrapod L-chain sequences were observed to form an orthologous group.

Additionally, a few teleost species including *S. salar*, *D. rerio* and *T. bernachii* appeared to possess several M-chain isoforms. The ferritin subunits isolated from *O. mykiss* were also observed to cluster with the M-chains, which suggest that these sequences previously have been erroneously described as H-chain isoforms [20].

### Codon substitution analysis

The *d_N_*/*d_S_* ratio were calculated from the estimated *d_N_* and *d_S_* values, to assess any effects of positive selection (*d_N_*/*d_S_*>1) on ferritin. The *d_N_*/*d_S_* ratio for the *S. salar* isoforms ranged from 0.0564 to 0.1181 (Table S2). Similarly, the *d_N_*/*d_S_* values for ferritin isoforms in *O. mykiss*, which is closely-related to *S. salar*, were found in the range of 0.111 to 01.1 As for the *d_N_*/*d_S_* between ferritin isoforms from other species, all of them were less than 0.3.

### Tissue distribution of ferritin isoforms

**Figure 4:**
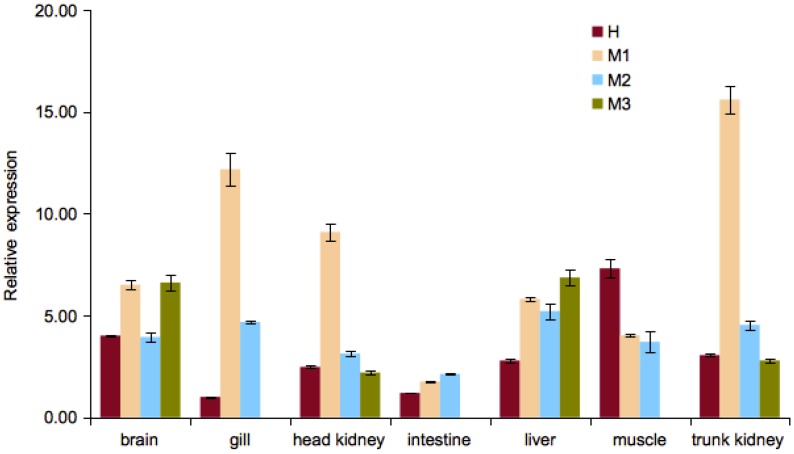
Tissue distribution of the ferritin H- and M-chain isoforms (H, M1, M2, M3) in *S. salar*. The relative expression of H-chain was measured as the total expression of the H1 and H2 isoforms. The relative expression of each isoform was normalised to the averaged expression of *ef1α* and *βact*. Bars represent standard errors mean (± SEM, n = 4).

The expression of the ferritin isoforms was examined in in 7 tissues (trunk kidney, head kidney, liver, muscle, brain, gill and intestine) from 4 healthy juvenile *S. salar* individuals. The relative expression of the ferritin isoforms was normalised to the expression of *ef1α* and *βact*, and presented as relative to the lowest ferritin expression value in a particular tissue (Figure 4). The expression of the H-chain (H1 and H2) was found in all of the examined tissues and the highest expression was observed in the muscle tissue. The expression of the M-chain isoforms (M1, M2 and M3) exhibited variation among the different tissues. M1 showed the highest expression in trunk kidney, followed by almost similar expression values in gills and head kidney. In contrast, the highest expression levels of M2 were almost similar among the liver, gills and trunk kidney. On the other hand, the expression of M3 was highest in liver and brain, but was not found in detectable levels in the gills, intestine and muscle (Ct>35) (data not shown).

### Modulation of ferritin expression following infection

**Figure 5:**
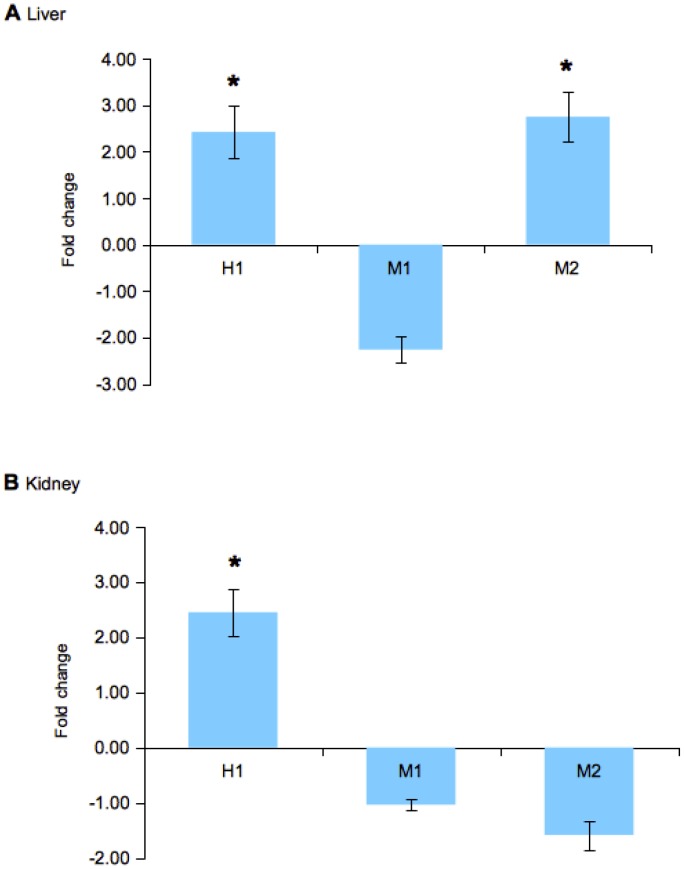
Fold changes in the expression of the ferritin H- and M-chain isoforms (H, M1, M2, M3) in the A. liver and B. kidney tissues of *S. salar* 24 hours post-infection with attenuated *A. salmonicida*. Bars represent standard errors mean (± SEM, n = 8) and asterisks indicate significant differences (p<0.05, *t*-test).

Total RNA was extracted from the liver and kidney tissues of *S. salar* at 24 hours post-infection with attenuated *A. salmonicida* to examine the expression changes of the ferritin isoforms. The expression of ferritin isoform M3 was excluded as several samples exhibited low peaks at Ct>35, which is generally considered unreliable due to the accumulation of background noise or non-specific fluorescence at that stage [40]. In general, distinct expression profiles were observed between the ferritin isoforms in the liver and kidney tissues in response to infection (Figure 5). In the liver, there was a significant increase of approximately 2.5-fold in the expression of the H-chain and M2. In contrast, the expression of M1 exhibited a minor decrease of 1.5-fold. The expression of the H-chain in the kidney also increased significantly by 2.5-fold, although the expression of M1 and M2 was decreased by approximately 1.5-fold.

### Ferritin expression in cell line stimulated with interleukin-1β

**Figure 6:**
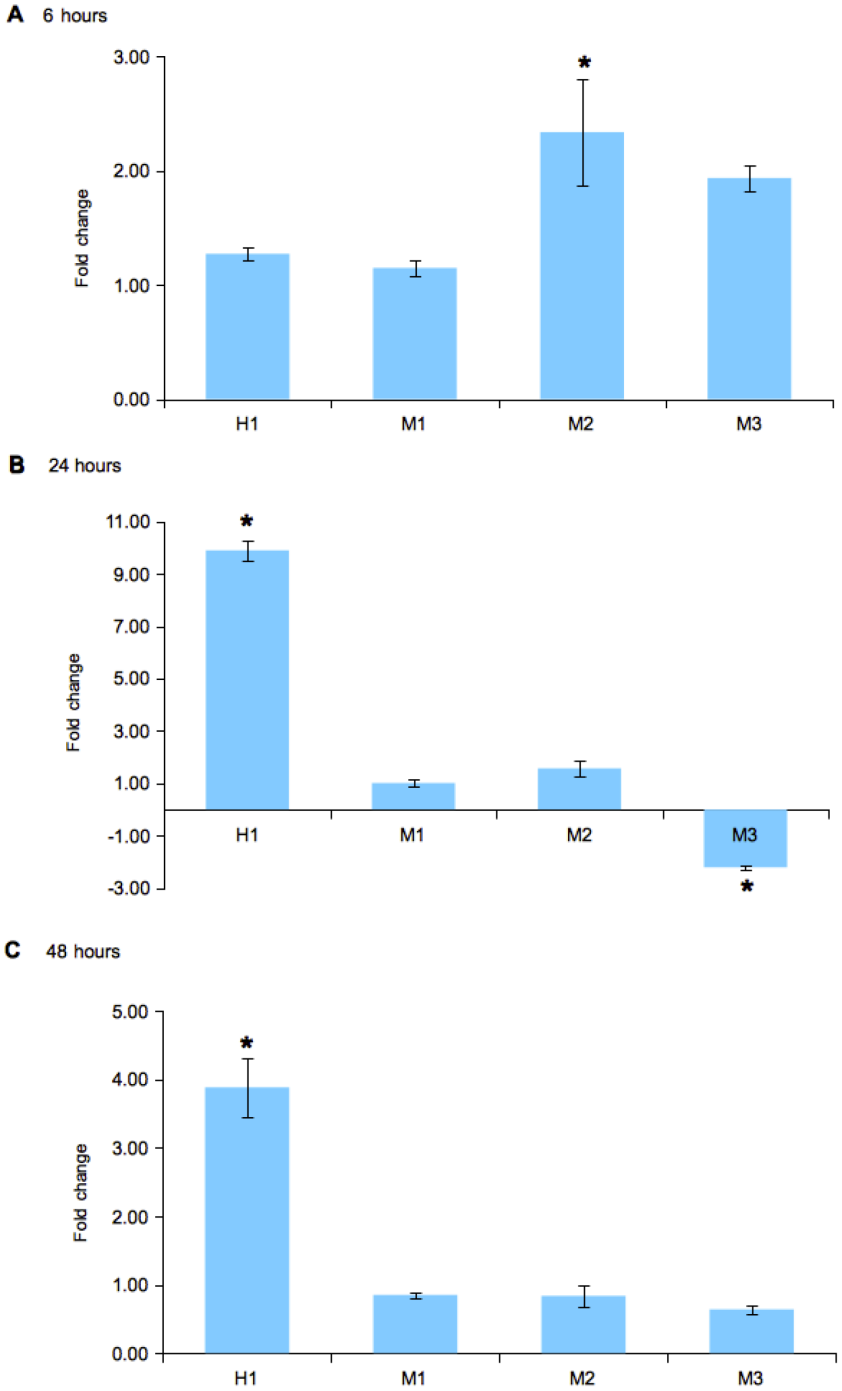
Fold changes in the expression of the ferritin H- and M-chain isoforms (H, M1, M2, M3) in *S. salar* muscle cell culture stimulated with IL-1β after A. 6 hour, B. 24 hours and C. 48 hours. Bars represent standard errors mean (± SEM, n = 4) and asterisks indicate significant differences (p<0.05, *t*-test).

The effects of IL-1β on ferritin expression were assessed using stimulated muscle cell culture. However, the M3 isoform was excluded from the final analysis due to several samples that exhibited Ct>35. Differential expression between the ferritin isoforms was observed at 6, 24, and 48 hours post-stimulation (Figure 6). The ferritin isoforms (H-chain, M1, M2) showed slight increases in expression at 6 hours post-stimulation, led by M2 that showed approximately 2-fold increase. At 24 hours post-stimulation, however, there was a significant 8.5-fold increase in H-chain expression. On the other hand, M3 exhibited 2-fold decrease in expression while the expression of M1 and M2 appeared relatively unchanged. At 48 hours, the expression of H-chain was increased by approximately 3-fold while M1 and M2 exhibited slight decreases in expression (less than 2-fold).

## Discussion

In this study, we report the characterisation of two H-chain and three M-chain ferritin isoforms from *S. salar*. Although multiple isoforms of a ferritin chain have been described previously (e.g. three H-chain isoforms in *O. mykiss*), to our knowledge our study is the first report of multiple isoforms of both H- and M-chains in a vertebrate species [20]. We further examined the distribution of the ferritin isoforms across tissues as well as the expression in infected fish and cytokine-stimulated muscle cells.

Our study is an expansion of the first reported teleostean ferritin that described the H- and M-chains in *S. salar* and the tissue distribution of both proteins [13]. Due to the presence of ferritin pseudogenes in vertebrates and high similarities between the ferritin chains, specific screening steps need to be carried out prior to the identification of novel ferritin sequences [12], [41]. In *S. salar*, we identified five ferritin sequences (H1, H2, M1, M2, M3) that possess IREs in the 5′-untranslated terminal, which is an acknowledged feature of vertebrate ferritins that are translationally-regulated by iron [42]. We then examined the gene organisation of those sequences and found that all five sequences share a 4-exon/3-intron structure, which is identical to the ferritin genes reported in other animals [8].

Our subsequent phylogenetic analysis of the ferritin sequences from *S. salar* and various vertebrate species shows a clear distinction between the H-chains and the M-/L-chains. The naming of the teleost M- and tetrapod L-chains was previously a source of confusion, and recently it has been suggested that both these proteins are orthologous [18]. The H-chain (Salsa_H) protein sequence in *S. salar* that was reported previously appeared to correspond with the H1 ferritin isoform that we isolated with 100% similarity (Fig. S2) [13]. However, there was no corresponding match between the M-chain isoforms M1, M2, M3 with the M-chain protein (Salsa_M) described in the earlier study as each isoform differed in at least two amino acid residues with the latter [13]. Notably, there were also no identical matches for Salsa_M in the current GenBank EST and genomic databases, in particular for the Arg15 residue in Salsa_M. Further studies would be needed to clarify if Salsa_M is indeed a distinct isoform apart from M1, M2, M3 and whether additional isoforms still exist in *S. salar*.

We also observed from our data mining and phylogenetic analysis that most teleost species possess multiple isoforms of the M-chain, in contrast to mammals that typically possess only a single copy of the L-chain gene. Additionally, *S. salar* is thus far the only teleost species that appears to possess more than one H-chain isoform (other than the ferritin isoforms in *O. mykiss* that appear to be incorrectly annotated) [20]. The presence of several ferritin isoforms in *S. salar* and *O. mykiss* could perhaps be attributed to the whole genome duplication event in teleost, as well as a subsequent duplication within the salmonid lineage [43], [44]. It would be interesting in the future to determine whether multiple H-chain isoform exists in *O. mykiss* as well. However, this hypothesis does not fully explain the reports of multiple M/L-chain isoforms in species that have not been reported to undergo additional rounds of duplication (e.g. *D. rerio*, *T. bernacchii*). It could therefore be a case where in species that exhibited possession of only one M-chain isoform (*Sciaenops ocellatus*, *P. crocea*, *S. maximus*, *T. newnesi*) in the phylogenetic tree, additional isoforms might exist and that the detection of these isoforms were complicated by spatio or temporal-specific expressions.

Despite the presence of multiple ferritin isoforms, our analysis of *d_N_*/*d_S_* ratio suggests that there is no positive selection as all the pairwise sequence *d_N_*/*d_S_* values were less than one. This observation was not wholly unexpected, as it is likely there is purifying selection on ferritin, which is extremely conserved protein found in almost all eukaryotes [1], [8]. Although there is a clear distinction between the H- and M/L-chains, clearly the core function of iron sequestration needs to be maintained. It is also important to note that the ferritin protein exists as a heterodimer of both chains; any changes to either chain could prevent proper formation and/or function of the heterodimer [8]. It is therefore likely that the changes in protein sequence of the ferritin chains or multiple M/L isoforms are minor tweaks to the iron sequestration process such as iron-binding affinity or iron-oxidation rate.

The distribution of the H-chain (measured as the combined expression of H1 and H2) in various tissues of the *S. salar* was noticeably distinct from the M-chain isoforms, in response to attenuated *A. salmonicida* (Figure 4). H-chain expression was highest in the muscle tissues, which was expected in line with its role in tissues that require rapid iron-exchange [8]. On the other hand, the expression of M1 was highest in trunk kidney, followed by gills and head kidney. As for M2, its highest expression levels were relatively similar between the liver, gills and trunk kidney. The expression of M3 was highest in the liver and brain, thought it was not detected in the gill, intestine and trunk kidney tissues. Further studies on M3 would be useful to determine whether its expression is limited to specific condition or in tissues that were excluded from this study.

The high expression of M2 in the liver is consistent with the role of the M-chain in iron-storage. However, the high expression of both M1 and M2 in both the kidney tissues (head and trunk kidney) is interesting as it was reported that the H-chain is the main subunit in mammalian kidney. Nevertheless, there is a major difference between teleostean and mammalian kidney, in terms of the kidney also functioning as the major haematopoietic organ in the absence of the bone marrow in teleosts [45]. In mammals, the L-chain was reported to be the highest expressed gene in macrophages that were activated by cytokines involved in haematopoiesis, and that the macrophages provide iron to maturing erythroblasts [46], [47]. As the M-chain and L-chain are orthologous, it will be useful to examine in greater detail the roles of the ferritin subunits in fish and mammals respectively [18].

The expression pattern of ferritin in the gills and intestine could be attributed to the physiology of both tissues in the context of nutritive iron uptake [48]. There is a significant intake of aquatic iron across the gills of freshwater fish and to a lesser degree, through intestinal absorption in marine fish [48]. Therefore, the moderate expression of M1 and M2 in the gills could be linked to iron uptake activity as the tissue samples were extracted from juvenile salmon that were kept in freshwater conditions. Similarly, this could also explain the extremely low expression of all ferritin isoforms in the intestine. It would be interesting in the future to follow up with an examination if a contrary pattern of expression is observed in the gills and intestines of adult salmons that dwell in a marine environment.

Our subsequent examination in salmons that were infected with attenuated *A. salmonicida* also found differential expression between the ferritin isoforms (Figure 5). The expression was measured from individuals that demonstrated a strong APR, as indicated by a large increase in the expression of serum amyloid A [35]. The significant increase of the H-chain in the liver and kidney tissues was consistent with observations in other teleost species such as *P. crocea* [15], *S. maximus* [16] and *Ictalurus punctatus* [49], suggesting a role for the H-chain during the APR

In contrast, the M-chain isoforms exhibited differential expression in the liver and kidney respectively. The expression of M1 was relatively unchanged in the liver and showed minor decrease in the kidney. On the other hand, M2 showed contrasting expression in both tissues – a slight decrease in the liver but a significant increase in kidney. This observation was particularly interesting as to our knowledge; previous studies in vertebrate ferritin have consistently reported increased ferritin expression during an APR [50], [51]. The different expression patterns displayed between the ferritin chains (H- and M-chains) as well as the individual isoforms (M1 and M2) appear to suggest the possibility of more distinctive and complex roles for ferritin during an immune response than previously assumed.

To further understand the regulation of ferritin expression, we analysed its expression in muscle cells stimulated with IL-1β, a major pro-inflammatory cytokine during an APR [52]. Stimulation with IL-1β displayed a small positive effect on the expression of all ferritin isoforms within 6 hours (Figure 6A). After 24 hours, the expression of the H-chain showed a significant 8.6-fold increase while the expression of the M1 and M2 was relatively unchanged (Figure 6B). Interestingly, the M3 isoform showed a significant 2.2-fold decrease in expression. However, post 48 hours the H-chain showed approximately 3.2-fold increase in expression while the expression of the M-chain isoforms were relatively unchanged (Figure 6C). Clearly, the differential expression patterns observed in our study strongly suggest that the expression of H-chain and M-chain isoforms during the APR are individually regulated by various enhancers and antagonistic factors.

Although further studies are necessary, it is possible that the existence of multiple ferritin isoforms in teleosts allows for increased dynamic control of iron storage in different cell types, in which each ferritin isoform (of H- and M-chains) possess varying degrees of iron affinity. An example of this can be observed in hepcidin, where the roles of iron regulation and antimicrobial activity are distinguished between the multiple hepcidin isoforms in teleosts [53]. Additionally, the potency of the anti-microbial activity varies between the hepcidin isoforms. Similarly, the distinct ferritin isoforms could be involved in accommodating the dynamic needs of iron in *S. salar* throughout different developmental stages and aquatic (freshwater/marine) environments.

## Conclusions

In this study, we isolated and characterised five novel ferritin isoforms (H1, H2, M1, M2, M3) from the *S. salar*. The identities of these isoforms as those belonging to the H- and M-chains were supported by prediction of the IRE and analysis of the gene organisation. Additional support of these ferritin isoform identities was provided through phylogenetic analysis on vertebrate ferritin, which highlights the need for careful annotation of ferritin subunits. We analysed the expression profiles of these ferritin isoforms, which exhibited distinct differences across tissues as well as changes in response to infection by *A. salmonicida* and stimulation with IL-1β. These unique expression profiles clearly show the importance of distinguishing between the various ferritin isoform, and that the roles of ferritin within the context of the immune response could be far more complex than previously assumed.

## Supporting Information

Figure S1 **The predicted IRE of the ferritin isoforms in** ***S. salar*** **(H1, H2, M1, M2, M3) based on identical cDNA sequences from GenBank.** The blue arrow indicates the apical UGC-bulge. The different nucleotide residues at the sixth position of the apical loop between the H- and M-chains are indicated by red boxes.
(TIF)

Figure S2 **Multiple sequence alignment of ferritin isoforms in** ***S. salar*** **(H1, H2, M1, M2, M3) and other vertebrates.** The conserved ferroxidase centres and nucleation sites are respectively indicated by green and blue shadings.
(PDF)

Table S1 **Accession numbers of sequences used in phylogenetic analysis.**
(PDF)

Table S2 **List of** ***d_N_/d_S_*** **values between ferritin isoforms in** ***S. salar*** **and** ***O. mykiss*** **.**
(PDF)
