# Supplementary figures and images for: Cloning and Characterisation of Multiple Ferritin Isoforms in the Atlantic Salmon (Salmo salar)
Source: PLoS One. 2014 Jul 31;9(7):e103729. doi: 10.1371/journal.pone.0103729 (PMC4117605; doi:10.1371/journal.pone.0103729)

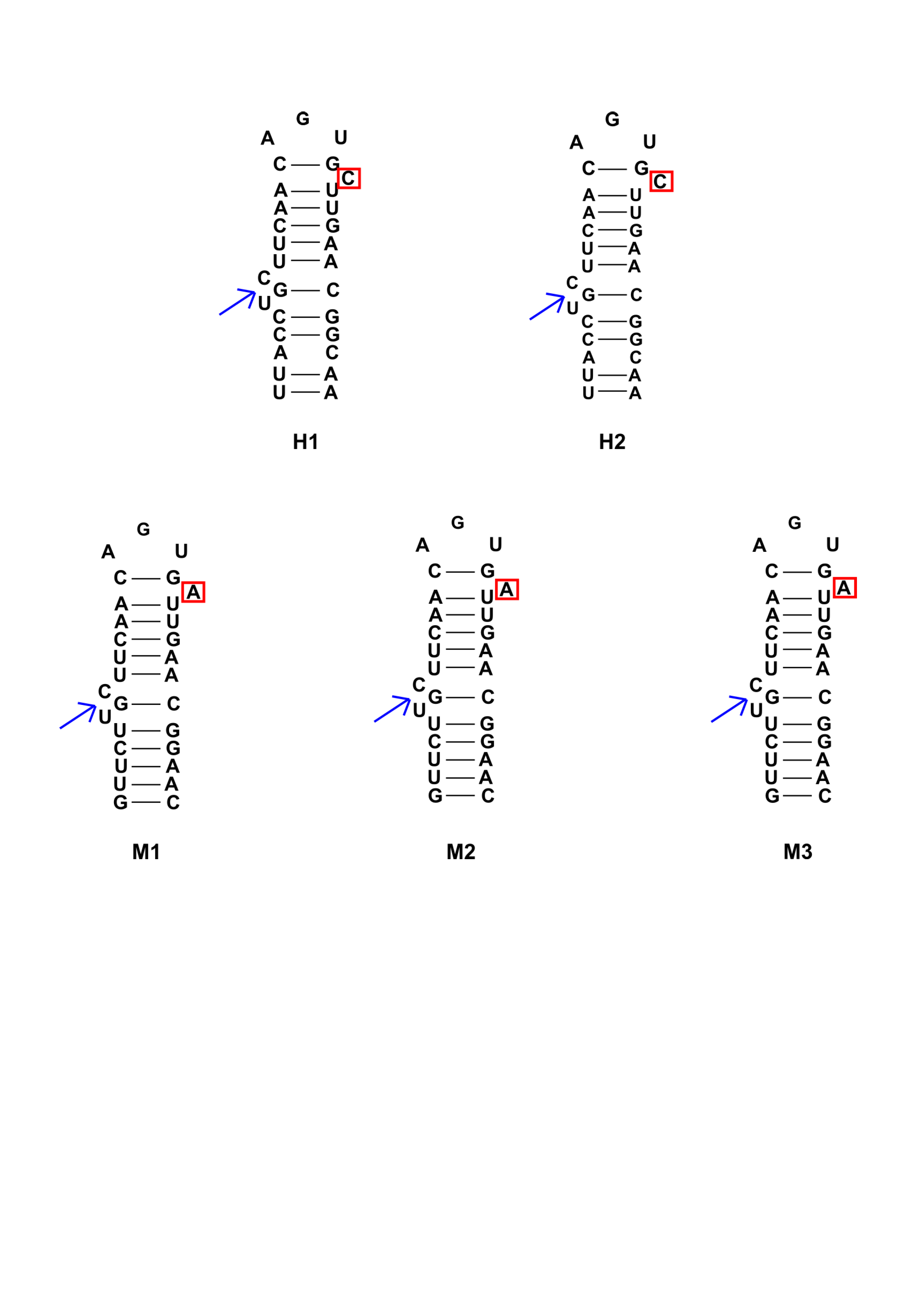

Supplement: Figure S1 — The predicted IRE of the ferritin isoforms in S. salar (H1, H2, M1, M2, M3) based on identical cDNA sequences from GenBank. The blue arrow indicates the apical UGC-bulge. The different nucleotide residues at the sixth position of the apical loop between the H- and M-chains are indicated by red boxes. (TIF) [file pone.0103729.s001.tif]
