# Supplementary material for: Cloning and Characterisation of Multiple Ferritin Isoforms in the Atlantic Salmon (Salmo salar)
Source: PLoS One. 2014 Jul 31;9(7):e103729. doi: 10.1371/journal.pone.0103729 (PMC4117605; doi:10.1371/journal.pone.0103729)
Supplement: Figure S2 — Multiple sequence alignment of ferritin isoforms in S. salar (H1, H2, M1, M2, M3) and other vertebrates. The conserved ferroxidase centres and nucleation sites are respectively indicated by green and blue shadings. (PDF) [file pone.0103729.s002.pdf]

Figure S2

```
                       ....|....| ....|....| ....|....| ....|....| ....|....| ....|....| ....|....| ....|....| ....|....| ....|....|
                               10         20         30         40         50         60         70         80         90        100
H1                     --M----TS- QVRQNFHQDC EAAINRQINL ELYASYVYLS M--AYYFDRD DQALHNFAKF FKNQSHEERE HAEKLMKVQN QRGGRIFLQD VKKPEKDEWG
H2                     --M----TS- QVRQNFHQDC EAAINRQINL ELYASYVYLS M--AYYFDRD DQALHNFAKF FKNQSHEERE HAEKLLTVQN QRGGRIFLQD VKKPEKDEWG
D. rerio 4             --M----SS- QVRQNFEEAC EAAVNRQINM ELYASYVYLS M--SYYFDRD DQALHNFAKF FRHQSHEERE HAEKLMKFQN QRGGRIFLQD VKKPEKDEWG
T. bernacchii H        --M----ES- QVRQNFHKDC EAAINRQINL ELYASYSYLS M--AYYFDRD DVALPGFAHF FKQQSEEERE HAEKLLKFQN QRGGRIFLQD VKKPDRDEWG
T. newnesi H           --M----ES- QVRQNFHKDC EAAINRQINL ELYASYSYLS M--AYYFDRD DVALPGFAHF FKHQSEEERE HAEKLMKQQN QRGGRIFLQD VKKPDRDEWG
D. labrax H            --M----SS- QVRQNFHQDC EAAINRQINL ELYASYVYLS M--AYYFDRD DQALHNFAKF FRNQSQEERE HAEKLMKVQN QRGGRIFLQD IRKPERDEWG
S. maximus H           --M----SS- QVRQNFHQDC EAAINRQINL ELYASYVYLS M--GYFFDRD DQALHNFAKF FRNQSHEERE HAEKLMKLQN QRGGRIFLQD IRKPERDEWG
P. crocea H            --M----SS- QVRQNFHQDC EAAVNRQINL ELYASYVYLS M--AYYFDRD DQALHNFAKF FRNQSQEERE HAEKLMKLQN QRGGRIFLQD IRKPERDEWG
S. salar H             --M----TS- QVRQNFHQDC EAAINRQINL ELYASYVYLS M--AYYFDRD DQALHNFAKF FKNQSHEERE HAEKLMKVQN QRGGRIFLQD VKKPEKDEWG
X. tropicalis 3        --M----NS- QVRQNYHQEC EAAINRQVNM ELYASYVYLS M--AYYFDRD DVALKNFSKY FLHQSHEERE HAEKLMKMQN QRGGRIFLQD VKKPDRDEWA
H. sapiens H           --MTTASTS- QVRQNYHQDS EAAINRQINL ELYASYVYLS M--SYYFDRD DVALKNFAKY FLHQSHEERE HAEKLMKLQN QRGGRIFLQD IKKPDCDDWE
M. musculus H          --MTTASPS- QVRQNYHQDA EAAINRQINL ELYASYVYLS M--SCYFDRD DVALKNFAKY FLHQSHEERE HAEKLMKLQN QRGGRIFLQD IKKPDRDDWE
R. norvegicus H        --MTTASPS- QVRQNYHQDS EAAINRQINL ELYASYVYLS M--SCYFDRD DVALKNFAKY FLHQSHEERE HAEKLMKLQN QRGGRIFLQD IKKPDRDDWE
B. taurus H            --MTTASPS- QVRQNYHQDS EAAINRQINL ELYASYVYLS M--SYYFDRD DVALKNFAKY FLHQSHEERE HAERLMKLQN QRGGRIFLQD IKKPDRDDWE
E. caballus H          --MTTAFPS- QVRQNYHQDS EAAINRQINL ELHASYVYLS M--SFYFDRD DVALKNFAKY FLHQSHEERE HAEKLMKLQN QRGGRIFLQD IKKPDQDDWE
M1                     --M----ES- QIRQNYHHDC EAAINRMINM EMFASYTYTS M--AFYFSRD DVALPGFAHF FKENSDEERE HADKLLSFQN KRGGRILLQD IKKPERDEWG
M2                     MKM----KS- QVRQNYHDDC EVAINRMINM EMFASYTYTS M--AFYFSRD DVALPGFAHF FKENSDEERE HADKLLSFQN KRGGRIVLQD IKKPERDEWG
M3                     MKI----ES- QIRQNYHDDC EVAINRMINM EMFASYTYTS M--AFYFSRD DVALPGFAHF FKENSEEERE HADKLLSFQN KRGGRILLQD ITKPESDEWG
D. rerio 1             --M----ETS QIRQNYARDS EAAINKMINL ELYAGYTYTS M--AHYFKRD DVALPGFAKF FKKNSEEERE HAEKFMEFQN KRGGRIVLQD IKKPDRDVWG
D. rerio 2             --M----DS- QVRQNYDRDC EALINKMINL ELYAGYTYTS MATAFYFDRD DVALPGFAKF FKKNSEEERE HAEKFMEFQN KRGGRIVLQD IKKPERDEWD
D. rerio 3             --M----ETC QIRQNYDSDC EALINKMINL ELYAGYTYTS M--AHYFKRD DVALPGFAKF FKNNSEEERE HAEKFMEFQN KRGGRIVLQD IKKPGRDVWD
D. rerio 5             --M----ETS QVRQNYARDS EAAINKMINL ELYAGYTYTS MASAHYFKRD DVALPGFAKF FNKNSEEERE HAEKFMEFQN KRGGRIVLQD IKKPDRDVWG
T. bernacchii M (spl)  --M----DS- QVRQNYHRDC EAAVNRMINM ELFASYSYTS M--AFYFSRD DVALPGFAHF FKENSDEERE HADKLLTFQN SRGGRIFLQD IKKPERDEWG
T. bernacchii M (liv)  --M----DS- QVRQNYHRDC EAAVNRMINM ELFASYSYTS M--AFYFSRD DVALPGFAHF FKENSDEERE HADKLLTFQN SRGGRIFLQD IKKPERDEWG
T. newnesi M           --M----DS- QVRQNYHRDC EAAVNRMINM ELFASYSYTS M--AFYFSRD DVALPGFAHF FKENSEEERE HADKLLTFQN SRGGRIFLQD IKKPERDEWG
S. maximum M           --M----ES- QVRQNYNRDC EAAVNRMVNM ELFASYTYTS M--AFYFSRD DVALPGFSHF FKENSEEERE HADKLLSFQN NRGGRIFLQD VKKPEKDEWG
P. crocea M            --M----ES- QVRQNYHRDC EAAINRMVNM ELFASYTYTS M--AFYFSRD DVALPGFSHF FKENSDEERE HAQKLLSFQN KRGGRIFLQD VKKPERDEWG
S. ocellatus M         --M----ES- QVRQNYHRDC EAAINRMVNM ELFASYTYTS M--AFYFSRD DVALPGFSHF FKENSDEERE HAQKLLSFQN KRGGRIFLQD VKKPERDEWG
O. mykiss H1           --M----ES- QIRQNYHHDC EAAINRMINL EMFASYTYTS M--AFYFSRD DVALRGFAHF FKENSDEERE HAEKLLSFQN KRGGRILLQD IKKPERDEWG
O. mykiss H2           --M----ES- QIRQNYHHDC EAAINRMINL EMFASYTYTS M--AFYFSRD DVALSGFAHF FKENSDEERE HADKLLSFQN KRGGRILLQD IKKPERDEWG
O. mykiss H3           --M----ES- QIRQNYHHDC EAAINRMINL EMFASYTYTS M--AFYFSRD DVALRGFAHF FKENSDEERE HADKLLSFQN KRGGRILLQD IKKPERDEWG
S. salar M             --M----ES- QIRQNYHHDC ERAINRMINM EMFASYTYTS M--AFYFSRD DVALPGFAHF FKENSEEERE HADKLLSFQN KRGGRILLQD IKKPERDEWG
X. tropicalis 1        --M----IS- QVRQNYSHDC EAAVNRMVNL EMYASYTYLS M--SHYFDRD DVALHHVAEF FKEQSKEERE CAEKLMKCQN KRGGRIVLQD IKKPERDEWG
X. tropicalis 2        MSA----QS- QIRQNYHEES EAGINRIANL ELQTSYVYLS L--GYYFDRD DVALSKFSKY YRELSEKKRD HAEDLLKFQN KRGGRVVLQD IKKPDADEWG
X. tropicalis 4        --M----QS- QVRQNFNSDC EAAINRMVNM ELYASYVYLS M--SYYFDRD DVALHHVAKF FKEQSHEERE HAEKFLKYQN KRGGRAVLQD IKKPERDEWG
H. sapiens L           --M----SS- QIRQNYSTDV EAAVNSLVNL YLQASYTYLS L--GFYFDRD DVALEGVSHF FRELAEEKRE GYERLLKMQN QRGGRALFQD IKKPAEDEWG
M. musculus L          --M----TS- QIRQNYSTEV EAAVNRLVNL HLRASYTYLS L--GFFFDRD DVALEGVGHF FRELAEEKRE GAERLLEFQN DRGGRALFQD VQKPSQDEWG
R. norvegicus L        --M----TS- QIRQNYSTEV EAAVNRLVNL HLRASYTYLS L--GFFFDRD DVALEGVGHF FRELAEEKRE GAERLLKLQN ERGGRALFQD VQKPSQDEWG
B. taurus L            --M----SS- QIRQNYSTEV EAAVNRLVNM QLRASYTYLS L--GFYFDRD DVALEGVGHF FRELAKEKRE GAERLLKLQN QRGGRALFLD VQKPSQDEWG
E. caballus L          --M----SS- QIRQNYSTEV EAAVNRLVNL YLRASYTYLS L--GFYFDRD DVALEGVCHF FRELAEEKRE GAERLLKMQN QRGGRALFQD LQKPSQDEWG
P. marinus             --M----TS- QVRQNYAQDV EAAINRQINM ELSASYVYLS M--AYYFDRD DVALNNFFKF FKEQSHEERE HAEKLMAYQN KRGGRVVLKD VKKPERDEWG
```

(continued)

```
                        ....|....| ....|....| ....|....| ....|....| ....|....| ....|....| ....|....| ....|....| ....|....| ....|.
                              110        120        130        140        150        160        170        180        190
H1                      SGVEALESSL QLEKSVNQSL LDLHKVCSEH NDPHMCDFIE THYLDEQVKS IKELGDWVTN LRRMGA---- ----PQNGMA EYLFDKHTLG KEST--
H2                      SGVEALESAL QLEKIVNQSL LDLHKVCSEH NDPHLCDFIE THYLDEQVKS IKELGDWVTN LRRMGA---- ----PQNGMA EYLFDKHTLG KEST--
D. rerio 4              SGVEALECAL QLEKSVNHSL LELHKLASQH NDPHMCDFIE THYLDEQVKS IKELGDHVTN LRRMGA---- ----PQNGMA EYMFDKLTLG KESS--
T. bernacchii H         SGLDALECAL QLEKNVNQSL LDLHKVCSEH NDPHMCDFLE THYLDEQVKS IKELGDWVTN LRRLGA---- ----PQNGMA EYLFDKHTLG K-----
T. newnesi H            SGLDALECAL QLEKNVNQSL LDLHKVCSEH NDPHMCDFLE THYLDEQVKS IKELGDWVTN LRRLGA---- ----PQNGMA EYLFDKHTLG K-----
D. labrax H             SGIEALECAL QLEKSVNQSL LDMHKLCSDH NDPHLCDFIE THYLDEQVKS IKELADWVTN LRRMGA---- ----PQNGMA EYLFDKHTLG KESS--
S. maximus H            SGVRLLNVAL QLEKSVNQSL LDLHKLCSDH NDPHMCDFIE THYLDEQVKS IKELADWVTN LRRMGA---- ----PKDGMA EYLFDKHTLG KESS--
P. crocea H             SGIEALECAL QLEKSVNQSL LDMHKLCSDH NDPHMCDFIE THYLDEQVKS IKELADWVTN LRRMGA---- ----PQNGMA EYLFDKHTLG KESS--
S. salar H              SGVEALESSL QLEKSVNQSL LDLHKVCSEH NDPHMCDFIE THYLDEQVKS IKELGDWVTN LRRMGA---- ----PQNGMA EYLFDKHTLG KEST--
X. tropicalis 3         NGLEALECSL QLEKSVNQSL LELHKLSTDH NDPHLCDFLE SHYLDEQVKS MKELGDHITN LRRMGA---- ----PSNGMA EYLFDKHTLG EHHD--
H. sapiens H            SGLNAMECAL HLEKNVNQSL LELHKLATDK NDPHLCDFIE THYLNEQVKA IKELGDHVTN LRKMGA---- ----PESGLA EYLFDKHTLG DSDNES
M. musculus H           SGLNAMECAL HLEKSVNQSL LELHKLATDK NDPHLCDFIE TYYLSEQVKS IKELGDHVTN LRKMGA---- ----PEAGMA EYLFDKHTLG HGDES-
R. norvegicus H         SGLNAMRCAL HLEKSVNQSL LELHKLATDK NDPHLCDFIE THYLNEQVKS IKELGDHVTN LRKMGA---- ----PESGMA EYLFDKHTLG HGDES-
B. taurus H             NGLTAMECAL CLERSVNQSL LELHKLATEK NDPHLCDFIE THYLNEQVEA IKELGDHITN LRKMGA---- ----PGSGMA EYLFDKHTLG HSES--
E. caballus H           NGLKAMECAL HLEKNVNESL LELHKLATDK NDPHLCDFLE THYLNEQVKA IKELGDHVTN LRRMGA---- ----PESGMA EYLFDKHTLG ECDES-
M1                      NGLEAMQCAL QLEKNVNQAL LDLHKIASDK VDPHLCDFLE THYLNEQVEA IKKLGDHITN LTKMDA---- ----VKNKMA EYLFDKHTLG GQS---
M2                      NGVEAMQCAL QLEKNVNQAL LDLHKIALDK VDPHLCDFLE THYLNEQVEA IKKLGDHITN LTKMDA---- ----VKNKMA EYLFDKHTLG GQS---
M3                      NGLEAMQCAL QLEKNVNQAL LDLHKIASDK VDPHLCDFLE THYLNEQVEA IKKLGDHITN LTKMDA---- ----VKNKMA EYLFDKHTLR GQS---
D. rerio 1              NGLIAMQCAL QLEKNVNQAL LDLHKLATEM GDPHLCDFLE THYLDEQVEA IKKLGDHITN LSKMDA---- ----GNNRMA EYLFDKQTL- -DS---
D. rerio 2              NGLTAMQCAL QLEKNVNQAL LDLHKVASQK GDPHLCDFLE SHYLNEQVEA IKKLGDHITN LSKMDA---- ----GNNRMA EYLFDKHTL- -DS---
D. rerio 3              NGLTAMQCAL QLEKSVNQAL LDLHKVASQK GDPHLCDLLE SHYLNEQVEA IKKLGDHITN LSKMDA---- ----GNNRMA EYLFDKHTL- -DS---
D. rerio 5              NGLIAMQCAL QLEKNVNQAL LDLHKLATEM GDPHMCEFLE THYLNEQVEA IKKLGDHITN LSKMDA---- ----GNNRMA EYLFDKHTL- -DS---
T. bernacchii M (spl)   NGVDVMQCAL QLEKNVNQAL LDLHKIASGK VDPHMCDFLE THYLNEQVES IKKLGDFITN LSRMDA---- ----VKNKMA EYLFDKHTMG GKN---
T. bernacchii M (liv)   SGLDALQSSL QLEKNVNQAL LDLHKIASDH TDPHMCDFLE THYLNEQVES IKKLGDFITN LSRMDA---- ----VKNKMA EYLFDKHTMG GKN---
T. newnesi M            SGLDALQSSL QLEKNVNQAL LDLHKIASDH TDPHMCDFLE THYLNEQVES IKKLGDFITN LSRMDA---- ----VKNKMA EYLFDKHTMG GKN---
S. maximus M            SGLEAMQCAL QLEKNVNQAL LDLHKLASDH VDPHMCDFLE THYLNEQVEA IKKLGDYISN LTRMDA---- ----KNNKMA EYLFDKHSLG GKS---
P. crocea M             SGLEAMQCAL QLEKNVNQAL LDLHKLASEH VDPHLCDFLE SHYLNEQVEA IKKLGDYISN LTRMDA---- ----HTNKMA EYLFDKHTLG GKS---
S. ocellatus M          SGLEAMQCAL QLEKNVNQAL LDLHKLASEH VDPHLCDFLE THYLNEQVEA IKKLGDYISN LSRMDA---- ----NTNKMA EYLFDKHSLG GKS---
O. mykiss H1            NGLEAMQCAL QLEKNVNQAL LDLHKIASGK VDPHLCDFLE THYLNEQVEA IKKLGDHITN LTKMDA---- ----VKNKMA EYLFDKHTLG GQS---
O. mykiss H2            NGLEAMQCAL QLEKNVNQAL LDLHKIASGK VDPHLCDFLE THYLNEQVEA IKKLGDHITN LTKMDA---- ----VKNKMA EYLFDKHTLG GQS---
O. mykiss H3            NGLEAMQCAL QLEKNVNQAL LDLHKIASDK VDPHLCDFLE THYLNEQVEA IKKLGDHITN LTKMDA---- ----VKNKMA EYLFDKHTLG GQS---
S. salar M              NGLEAMQCAL QLEKNVNQAL LDLHKIASDK VDPHLCDFLE THYLNEQVEA IKKLGDHITN LTKMDA---- ----VKNKMA EYLFDKHTLG GQS---
X. tropicalis 1         STLDAMQTAL DLEKHVNQAL LDLHNLATER KDPHICDFLE SEHLDEQVKH MKKFGDHITN LKRLGV---- ----PQNGMG EYLFDKHSLS ------
X. tropicalis 2         NGTKAMEVAL NLEKSVNQAL LDLHKIATDH ADPHMCDYLE REFLEEEVKI IKKLGDHLTN LKRVKA---- ----AEDGMG EYLFDKLTLG DSD---
X. tropicalis 4         NTLEAMQAAL QLEKTVNQAL LDLHKLASDK TDPHLCDFLE SEYLEEQVKA MKELGDYITN LKRLGV---- ----PQNGMG EYLFDKHTLG ESS---
H. sapiens L            KTPDAMKAAM ALEKKLNQAL LDLHALGSAR TDPHLCDFLE THFLDEEVKL IKKMGDHLTN LHRLGG---- ----PEAGLG EYLFERLTLK HD----
M. musculus L           KTQEAMEAAL AMEKNLNQAL LDLHALGSAR TDPHLCDFLE SHYLDKEVKL IKKMGNHLTN LRRVAGPQPA QTGAPQGSLG EYLFERLTLK HD----
R. norvegicus L         KTLEAMEAAL ALEKNLNQAL LDLHALGSAR TDPHLCDFLE SHFLDKEVKL IKKMGNHLTN LRRVAGPQPA QTGVAQASLG EYLFERLTLK HD----
B. taurus L             KTQDAMEAAL LVEKNLNQAL LDLHGLASAR GDPHICDFLE NHFLDEEVKL IKKMGDHLTN LRRLAG---- ----PQAGLG EYLFERLTLK HD----
E. caballus L           TTLDAMKAAI VLEKSLNQAL LDLHALGSAQ ADPHLCDFLE SHFLDEEVKL IKKMGDHLTN IQRLVG---- ----SQAGLG EYLFERLTLK HD----
P. marinus              SGLEAVQLAL QLEKNVNQSL LDLHALGSKE NDPQLCDFLE SHYLEEQVEA IKKLGDFVTN LKRLGA---- ----SQSGMG EYLFDKLTLG SSS---
```
