# Supplementary material for: Cloning and Characterisation of Multiple Ferritin Isoforms in the Atlantic Salmon (Salmo salar)
Source: PLoS One. 2014 Jul 31;9(7):e103729. doi: 10.1371/journal.pone.0103729 (PMC4117605; doi:10.1371/journal.pone.0103729)
Supplement: Table S1 — Accession numbers of sequences used in phylogenetic analysis. (PDF) [file pone.0103729.s003.pdf]

Supplementary Table 1: List of sequences used in phylogenetic analysis

**Species ***	**Accession number**
Danio rerio (E)	ENSDARG00000079938
	ENSDARG00000031776
	ENSDARG00000078342
	ENSDARG00000015551
	ENSDARG00000073936
Gasterosteus aculeatus (E)	ENSGACP00000007931
	ENSGACP00000007905
	ENSGACP00000020445
Tetraodon nigroviridis (E)	ENSTNIG00000011144
	ENSTNIG00000004260
Takifugu rubripes (E)	ENSTRUG00000014292
	ENSTRUG00000017266
Oryzias latipes (E)_	ENSORLG00000016761
	ENSORLG00000005872
Oreochromis niloticus (E)	ENSONIG00000006516
	ENSONIG00000002989
Xiphophorus maculatus (E)	ENSXMAG00000008596
	ENSXMAG00000018294
Salmo salar (U)	P49946
	P49947
Trematomus bernacchii (U)	P85838
	P85836
	P85839
Trematomus newnesi (U)	P85837
	P85835
Dicentrarchus labrax (G)	ACN80998.1
Scophthalmus maximus (G)	ADI24353.1
	ADI24354.1
Pseudosciaena crocea (G)	ACY75475.1
	ACY75476.1
Sciaenops ocellatus (G)	ADF80517.1
Oncorhynchus mykiss (G)	NP_001118019.1
	NP_001118020.1
	NP_001118021.1
Xenopus tropicalis (E)	ENSXETG00000021234
	ENSXETG00000006737
	ENSXETG00000021237

	ENSXETG00000021236
Taeniopygia guttata (E)	ENSTGUG00000005949
	ENSTGUG00000015659
Homo sapiens (U)	P02794
	P02792
Mus musculus (U)	P09528
	P29391
Rattus norvegicus (U)	P19132
	P02793
Bos taurus (U)	O46414
	O46415
Equus caballus (U)	Q8MIP0
	P02791
Canis familiaris (U)	Q95MP7
	Q53VB8
Sus scrofa (E)	ENSSSCP00000003417
	ENSSSCP00000026601
Petromyzon marinus (U)	AAN63033

*Database source: E – Ensembl, U – UniProt/SwissProt, G – GenBank
