# Supplementary material for: Cloning and Characterisation of Multiple Ferritin Isoforms in the Atlantic Salmon (Salmo salar)
Source: PLoS One. 2014 Jul 31;9(7):e103729. doi: 10.1371/journal.pone.0103729 (PMC4117605; doi:10.1371/journal.pone.0103729)
Supplement: Table S2 — List of dN/dS values between ferritin isoforms in S. salar and O. mykiss . (PDF) [file pone.0103729.s004.pdf]

Supplementary Table 2: List of d_N/d_S values between ferritin isoforms in *S. salar* and *O. mykiss*.

Sequence pair	d_N/d_S
H1 – H2	0.1364
H1 – M1	0.0915
H1 – M2	0.0894
H1 – M3	0.1002
H2 – M1	0.1181
H2-M2	0.0862
H2-M3	0.0848
M1-M2	0.0564
M1-M3	0.0525
M2-M3	0.1066
O.mykiss H1- O.mykiss H2	0.1499
O.mykiss H1 - O.mykiss H3	0.047
O.mykiss H2 - O.mykiss H3	0.0412
